# Funding the Way to Open Access

**DOI:** 10.1371/journal.pbio.0030097

**Published:** 2005-03-15

**Authors:** Robert Terry

## Abstract

The Wellcome Trust - the UK's largest non-governmental funder of biomedical research - is taking action to ensure the work it supports is available to all

Imagine this scenario. You're the director of one of the world's largest medical research charities, and you receive notification from one of your funded investigators in Africa reporting some exciting progress toward the development of a vaccine for malaria. The work has just been published, so you log onto the Web to do a quick keyword search, and a link to the article is brought up on your screen.

Then imagine the frustration when you click on the link to read the message, “Access Denied—access to this journal is restricted to registered institutional and individual subscribers.”

And there's the rub: this actually happened to the Director of the Wellcome Trust. Prior to this, the committee that advises the Wellcome Trust Library were already asking whether the Trust should adopt a formal position on the continually increasing prices of journal subscriptions and the problems this trend was causing research libraries.

These events encouraged the Trust to investigate the publication of scientific research, to see if there was anything research-funding organisations could be doing to stimulate change in what appears to be a failing market. As it turns out, there is quite a lot. I now believe it is the funders of research—charities, governments, and other publicly funded bodies such as national research agencies—who hold the purse strings that can untie scientific discoveries from a publishing market that is no longer serving the community as well as it could. That is why today the Trust is a leading advocate for enabling free access to research literature through support for new publishing models, such as that of the Public Library of Science, and the establishment of publicly accessible repositories, working in partnership with the United States National Institutes of Health–funded PubMed Central [1].

**Figure:**
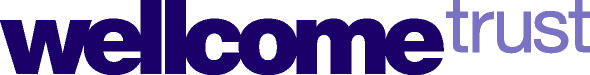


It is worth noting that the Trust is not a novice in seeking better ways to disseminate research findings. The fact that the sequence of the human genome is an openly accessible work is due in large measure to the Trust's determination that this information be in the public domain and not hidden behind commercial subscriptions. As a consequence of that insistence, we believe, these data are a more widely used and valuable resource.

“Trust-funded researchers will have to deposit an electronic version of their manuscripts in PMC to be made available for free via the Internet within 6 months of publication.”

The Trust began its investigation of the scientific publishing sector by commissioning two pieces of research: one to inform itself of the economics of the publishing sector, and a second to explore whether there were alternative business models out there that could enable research to have the quality assurance it needs (peer review) whilst being available for free, using the Web as the medium of publication.

## The Economics of Publishing

The first Trust-commissioned study described how scientific research publishing has traditionally worked and why it can be described, in economic terms, as a failing market [2]. Essentially, the producers (researchers as authors) and the consumers (researchers as readers) are isolated from any of the costs within the system. Researchers give away the copyright to their work, for free, to the publishers, who organise the peer review and copyedit the article. The publishers then sell it to libraries at prices that range from enough to cover their costs through to some pretty high profits—some over 30%. These profits escape from an otherwise self-contained financial cycle to satisfy shareholders or run learned societies; unlike typical publishing relationships, none are returned directly to the author (the researcher who wrote the piece) or even to the consulting experts (the researchers who provided the peer review).

At the same time, researchers as readers access the material, if they are able to do so, through their employing institution, either using the library or—more typically now—via the Internet through the institution's subscription. To the researcher this access appears free, effectively creating a market system that has no pressures from the producers or consumers to change. One consequence of this is that publishers have been able to increase subscription prices well above inflation; the United Kingdom has seen subscription rates rise by more than 200% in the last ten years (Blackwell's periodical price indexes; [3]). The money used to fund UK libraries is all public money, and over 90% of the funds paying for research in the UK university system is either government or charitable [4]—so in a sense the people who are paying for the research cannot access its findings without paying an additional fee.[Fig pbio-0030097-g002]

**Figure pbio-0030097-g002:**
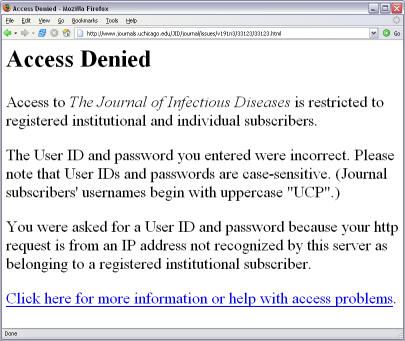
Access denied at *The Journal of Infectious Diseases*

This then begs the question of what alternatives there are to this traditional system, now that the Internet has become the researcher's tool of choice for searching and accessing the literature. The second piece of research commissioned by the Trust looked at different business models for research publishing, in order to address this question [5]. It compared open-access journals, which often levy a charge to publish but provide the journal for free, and the majority of the traditional models, which take the research for free but charge readers to read it.

This study convinced the Trust that the best way forward to improve access to research findings would be through open access to scientific research articles. This essentially means two things: first, that the copyright holder or holders must grant to the public a free, irrevocable, perpetual license to use, copy, distribute, and make derivative works of their research article, in any medium for any purpose (excepting those that constitute plagiarism or other dishonest acts, of course); and second, that a digital copy must be deposited in an open public archival repository (for example, the US National Library of Medicine's PubMed Central). Whilst a debate continues as to the most appropriate route to achieve open access to all research literature, it is important to bear in mind that the publication and the archiving of research articles are intrinsically linked. Both aspects of open access need to be explored and experimented with, and the Trust is actively pursuing solutions for the problems of both.

## Alternative Business Models

The findings of the second report seem to have caused quite a controversy—particularly in the suggestion that moving wholesale to an open-access publishing model might produce savings of up to 30% [6]. One common misinterpretation of this conclusion is that any such savings would be due solely to discontinuing the printed versions of publications that are freely available online. This is incorrect. In fact, if savings are to be made in an open-access model, they will largely be found in the variable costs of journal production—since an open-access journal will not have to cover the costs of subscription management, licence negotiations, or sales, and little is required for marketing and distribution.

In a comparison included in the report, an article in a good- to high-quality journal produced in the subscription model is estimated to cost US$2,750. The equivalent cost under an author-side payment model is estimated as US$1,950—a comparable saving of 30% on the costs, and a saving of 90% when the variable costs are compared. It must be remembered that cost does not equate to price, so to these figures, regardless of the mode of publication, must be added overhead expenses and, of course, profit. However, if a truly competitive market is created—where payments are directed to publishers not by third parties but by those directly involved in the scientific enterprise, who could easily compare the varying article processing charges of different open-access publishers—then the actual savings might well be substantially higher.

At its essence though, the open-access debate is not about economics, it is about access. That is why the Trust has been in discussion with the US National Library of Medicine about the possibility of creating a UK PubMed Central (UKPMC) as a publicly accessible repository for Trust-funded research.

## UK PubMed Central

The proposal is that a UKPMC will be run as a proper electronic library: it will collect, collate, and archive whole journals and be developed to receive single articles as well. Submission will be as straightforward as attaching a document to an email. UKPMC will be able to accept manuscripts in any format, including Microsoft Word, and it will be the responsibility of UKPMC to convert the files it receives into extensible markup language (XML) to enable the appropriate document type definition (DTD) to be assigned. UKPMC will also correct the structural, content, and consistency errors that occur when converting text for digital preservation, and provide the conversion process to print a “clear” PDF version of included articles to those users who download them. This is a process well used by the National Library of Medicine, and the one most suited for the long-term, digital preservation of articles.

And once articles are in a digital format they can be searched and used in different ways. For example, genome sequence data, chemical compounds, or protein structures embedded within an article can be searched for in other articles and linked directly to genome or structural databases uncovering new genetic markers, drug uses, or protein functions. The articles themselves become live research material greatly improving the efficacy of the research itself.

For a funder, having all its research in one format, “under one roof”, and searchable will improve the efficiency of strategy setting—for example, setting funding priorities—assessing the outputs of the funded research, and even gaining an insight into the impact of the work. As grants management becomes more electronic, there can be a direct link between original research proposals and the research outputs.

For a medical charity like the Trust, I believe it is our duty to actively encourage the most efficient processes available to maximise the likelihood that the research we fund will have the greatest possible health benefit.

That is why the Trust will be making it a requirement of its grant conditions that Trust-funded researchers deposit an electronic version of their manuscripts in UKPMC to be made available for free via the Internet within 6 months of publication. The delay means that this is not open access in the truest sense. However, the Trust considers that the development of a PubMed Central portal in the UK offers the best next step in the transition towards a situation where all high-quality peer-reviewed research is available for free via the Internet, whilst leaving all publishers room for manoeuvre in this changing market.

## Abbreviations

UKPMC: United Kingdom PubMed Central
